# Effect of Hydrogenated, Liquid and Ghee Oils on Serum Lipids Profile

**Published:** 2010

**Authors:** Noushin Mohammadifard, Masoud Nazem, Gholam-Ali Naderi, Faezeh Saghafian, Firoozeh Sajjadi, Maryam Maghroon, Ahmad Bahonar, Hasan Alikhasi, Fatemeh Nouri

**Affiliations:** 1 MSc, Department of Nutrition, Isfahan Cardiovascular Research Center, Isfahan University of Medical Sciences, Isfahan, Iran; 2 Associate Professor, Department of Surgery, School of Medicine, Isfahan University of Medical Sciences, Isfahan, Iran; 3 Associate Professor, Department of Basic Sciences, Isfahan Cardiovascular Research Center, Isfahan University of Medical Sciences, Isfahan, Iran; 4 BSc, Department of Nutrition, Isfahan Cardiovascular Research Center, Isfahan University of Medical Sciences, Isfahan, Iran; 5 MD, MPH, Isfahan Cardiovascular Research Center, Isfahan University of Medical Sciences, Isfahan, Iran; 6 Biostatistics Unit, Isfahan Cardiovascular Research Center, Isfahan University of Medical Sciences, Isfahan, Iran

**Keywords:** Serum lipids, Apoproteins, Liquid oil, Hydrogenated oil, Ghee, Clinical trial

## Abstract

**BACKGROUND:**

Trans fatty acids are known as the most harmful type of dietary fats, so this study was done to compare the effects of hydrogenated, liquid and ghee oils on serum lipids profile of healthy adults.

**METHODS:**

This study was a randomized clinical trial conducted on 129 healthy participants aged from 20 to 60 years old who were beneficiaries of Imam-e-Zaman charitable organization. Subjects were randomly divided into 3 groups and each group was treated with a diet containing cooking and frying liquid, ghee, or hydrogenated for 40 days. Fasting serum lipids, including total cholesterol (TC), triglyceride (TG), LDL-cholesterol (LDL-C), HDL-cholesterol (HDL-C), apoprotein A (Apo A), and apoprotein B (Apo B) were measured before and after the study.

**RESULTS:**

TC, TG and Apo B had a significant reduction in the liquid oil group compared to the hydrogenated oil group. In the ghee group TG declined and Apo A increased significantly (P < 0.01). Liquid oil group had a significant reduction in HDL-C, compared to the ghee oil group (P < 0.05).

**CONCLUSION:**

It was concluded that consuming liquid oil along with frying oil caused to reduce all serum lipid levels. However, ghee oil only reduced TG and increased HDL-C levels.

## Introduction

According to epidemiological and clinical studies, diets affect the occurrence, progress and prevention of non-communicable diseases, including cardiovascular diseases (CVD), cancers, diabetes and hypertension.^1^–^8^ Among the most important dietary interventions to prevent non-communicable diseases are the reduction of energy consumption, saturated fatty acids (SFAs) and trans fatty acid (TFAs), and the increment of unsaturated fats intake. Fats are consumed in foods in order to provide energy, essential fatty acids and fat soluble vitamins.^9^ In the last few decades, the reduction of fat intake has been the major recommendation for decreasing CVD risk.^10^ There is a great deal of evidence confirming that the type of dietary fat is more determinant in CVD development than its amount.^11^ SFAs cause an increase in serum total cholesterol (TC) and LDL-cholesterol (LDL-C) levels.^12^

It was especially observed that SFAs containing 12–16 carbon atoms which increase LDL-C, while stearic acid (C18:0) does not raise plasma cholesterol levels.^13^ In the past, the effects of fats on increasing plasma TC levels were estimated by their saturation degree;^14^, ^15^ however, the evidence obtained during this past decade indicates that the TFA existing in hydrogenated oils not only increases LDL-C, TC and apoprotein (Apo) B levels but also decreases HDL-C and Apo A levels,^16^ and therefore, TFAs are more harmful than SFAs.^17^ Scientists at Public Health School of Harvard University estimated that in the US in 2001, about 30,000 people died of CVD events caused by TFAs.^18^ Hydrogenated oils are the major source of TFA intake.^19^ Bahrami et al study reported that the mean of TFAs content of hydrogenated oils produced in Iran was 34.6 ± 6.6% (Range: 22.5–46.2%),^20^ which is much higher than the World Health Organization recommendation.^21^ Some studies have mentioned that ghee, which is produced from milk by traditional methods and is usually called "yellow oil" or "Kermanshahi oil", although contains high amounts of SFAs and cholesterol, is useful for decreasing LDL-C and increasing HDL-C.^22^, ^23^ There are some controversies about how ghee consumption and serum lipid profile are linked.^24^, ^25^ However, there is no precise scientific information in human subjects in this issue. In a study conducted on animal subjects in Iran, it's been observed that ghee oil consumption significantly increased HDL-C level but did not have any significant effect on other serum lipids.^26^ As TFAs are considered as the most harmful dietary fats, this study was conducted aiming to compare the effects of liquid and ghee oils with hydrogenated oil as a main source of TFA.

## Methods and Materials

### Study design and sampling

This randomized clinical trial has been conducted on 150 healthy subjects aged 20–60 years. They were chosen from beneficiaries of Imam-e-Zaman charitable organization and who consumed only hydrogenated oil in their diet. The normotensive, non-diabetic participants without cardiovascular diseases were invited to the study center. After fasting over night, venous blood samples were drawn at 7:00 and 10:00 am. Subjects with TC ≥ 240 mg/dl, TG ≥ 400 mg/dl, LDL-C ≥ 160 mg/dl or HDL-C ≥ 40 mg/dl, who also had body mass index (BMI) ≥ 35 were excluded. Then they were equally divided into three groups of hydrogenated, liquid and ghee oils. However, 21 were leaved out the study due to traveling, sickness, not willing to participate in the next sampling, or not complying with dietary recommendations. Therefore, 129 healthy subjects were included in the study finally.

After signing informed written consent, the subjects were referred to the trained nutritionist to obtain socio-demographic characteristics, past medical history and food habits by 24-hour recall questionnaire. Anthropometrical measurements were taken with shoes removed and the participants wearing light clothing.^27^ BMI was calculated by dividing the weight in kilograms to the square of height in meters.^27^ Eligible subjects who were consumed hydrogenated oil based on obtained food habits were randomly divided into 3 groups and they were taken a diet consultation with the same amount of oil containing cooking and frying liquid, ghee or hydrogenated oils, for 40 days.^28^ Ghee was provided from Bakhtiari nomads. In order to keep the type of oils similar among each group, oils were given to the subjects (every 10 days) by the project conductor. Dietary recommendations were done to the subjects by the same dietitian, so that the only difference among the three groups was the kind of dietary oil. Subjects were followed by phone every two weeks or in their referral to the study center for taking their oil.

### Biochemical measurements

Blood samples were taken while the subject had been fasting for 14 hours. Serum lipids levels, including TC, TG and HDL-C levels, were measured. TC and TG were determined by standard enzymatic method using special kits in Hitachi auto-analyzer. HDL-C was measured enzymatically after precipitating the other lipoproteins with dextran sulphate magnesium chloride.^29^ LDL-C was calculated by using of the Friedewald formula. Direct measurement of LDL-C was performed with a turbidimetric method for those with TG ≥ 400 mg/dl.^30^ Apoproteins A and B levels were determined by Merk kits.^31^ Blood samples were collected before and after the study,^32^ at Isfahan Cardiovascular Research Center laboratory, which meets the criteria of the National Reference Laboratory, a WHO-collaborating center.

### Statistical analysis

In the beginning of the study, the mean of age, BMI and serum lipids levels among the three groups was compared by one-way analysis of variance (ANOVA) test; comparison of the frequency distribution was conducted using chi-square test based on gender, education level and marital status. Mean of serum lipids levels before and after the study were compared by paired t-test in each group. The comparison of changes in serum lipids and Apo A and B levels between 3 groups was done with two-way ANOVA test by adjusting for age and gender.

## Results

The study included 32, 50 and 44 subjects in hydrogenated, liquid and ghee oil groups, respectively. They included 99 men and 34 women with the mean age of 34.1 ± 11.2 years. As shown in table 1, there is no significant difference in mean of age, serum lipids including TC, TG, LDL-C, HDL-C, Apo A and Apo B levels and also gender, educational and marital status distribution between 3 groups in the beginning of the study. Table 2 demonstrates the comparison between mean of serum lipids and Apo A and B levels before and after the study in each group.

**Table 1:** Basic characteristics and serum lipids in the beginning of the study

	Hydrogenated oil	Liquid oil	Ghee oil	P value
	
	Mean (SD)	Mean (SD)	Mean (SD)	
Number	37	50	44	NS*
Age (Y)	32.4 (11.3)	33.9 (11.5)	35.4 (10.9)	NS
Body mass index (Kg/m^2^)	26.5 (4.4)	26.1 (5.3)	25.7 (4.6)	NS
Total cholesterol (mg/dl)	174.9 (23.4)	176.7 (25.4)	183.8 (30.9)	NS
Triglyceride (mg/dl)	134.1 (52.8)	127.4 (52.1)	125 (52)	NS
HDL-C (mg/dl)	44.1 (7.5)	45.3 (6.9)	44 (5.6)	NS
LDL-C (mg/dl)	104.7 (19.5)	105.9 (22.6)	114.8 (27.2)	NS
Apoprotein A (mg/dl)	129.9 (17.3)	136.5 (17.4)	122 (14)	NS
Apoprotein B (mg/dl)	92.3 (17.9)	100.7 (23.5)	98.4 (20.1)	NS
**Gender**	**Frequency (%)**			

Male	10 (29.4)	12 (22.2)	12 (26.7)	NS
Female	24 (70.6)	42 (77.8)	33 (73.3)	NS
**Education level**				
Literacy	13 (38.2)	19 (35.2)	7 (15.6)	
Primary and guidance school	18 (52.9)	30 (55.6)	31 (68.9)	NS
High school and university	3 (8.8)	5 (9.3)	7 (15.6)	
**Marital status**				
Single	11 (32.4)	15 (27.8)	10 (22.2)	
Married	20 (25.6)	31 (39.7)	27 (34.6)	NS
Died and divorced	3 (15.8)	8 (42.1)	8 (42.1)	

* NS: Non significant

**Table 2:** Comparison of serum lipid before and after the study

	Before	After	P value
	
	Mean (SD)	Mean (SD)	
**Hydrogenated oil**			
Total cholesterol (mg/dl)	174.9 (23.4)	178.6 (25.3)	< 0.04
Triglyceride (mg/dl)	134.1 (52.8)	137.5 (51.8)	NS*
HDL-C (mg/dl)	44.1 (7.5)	43.5 (7)	NS
LDL-C (mg/dl)	104.7 (19.5)	107.1 (21.2)	NS
Apoprotein A (mg/dl)	129.9 (17.3)	125.8 (13.3)	< 0.03
Apoprotein B (mg/dl)	92.3 (17.9)	95.7 (21)	NS
**Liquid oil**			
Total cholesterol (mg/dl)	176.7 (25.4)	173.1 (26.2)	< 0.001
Triglyceride (mg/dl)	127.4 (42.1)	122.8 (41.6)	< 0.001
HDL-C (mg/dl)	45.4 (6.9)	44.3 (6.8)	NS
LDL-C (mg/dl)	105.9 (22.6)	104.3 (24.4)	NS
Apoprotein A (mg/dl)	126.5 (17.4)	127.8 (14.8)	NS
Apoprotein B (mg/dl)	100.7 (23.5)	94.1 (22.6)	< 0.01
**Ghee oil**			
Total cholesterol (mg/dl)	183.8 (30.9)	183.5 (28.6)	NS
Triglyceride (mg/dl)	125 (52)	122.6 (49.3)	NS
HDL-C (mg/dl)	44 (5.6)	45.3 (7.6)	NS
LDL-C (mg/dl)	114.8 (27.2)	114.1 (24.5)	NS
Apoprotein A (mg/dl)	122 (14)	125.4 (13)	< 0.001
Apoprotein B (mg/dl)	98.4 (20.1)	98.7 (17.3)	NS

* NS: Non significant

In the hydrogenated oil group, TC increased and Apo A decreased significantly (P < 0.05). Liquid oil group had a significant reduction in TC, TG, and Apo B (P < 0.01) and in the ghee group, Apo A significantly increased.

**Figure 1:**
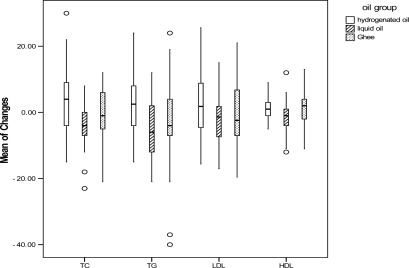
Comparing the mean of serum lipids charges between hydrogenated, liquid and ghee oil groups

**Figure 2:**
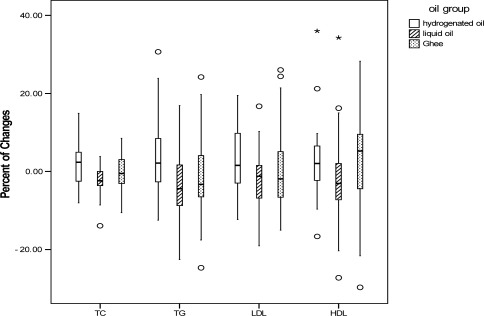
Comparing the percent of serum lipids changes between hydrogenated, liquid and ghee oil groups

**Figure 3:**
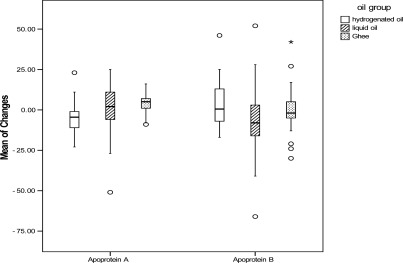
Comparing the mean of apoproteins changes, between hydrogenated, liquid and ghee oil groups

**Figure 4:**
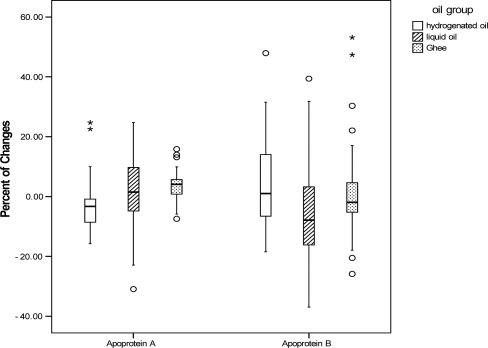
Comparing the percent of apoproteins changes between hydrogenated, liquid and ghee oil groups

Except for LDL-C, and Apo A and B levels, the comparison of the mean and percentage of serum lipids, changes with age and sex adjustment, revealed a significant difference among the three studied groups (Figure 1–4). TC, TG and Apo B levels had a significant reduction in liquid oil group when compared with hydrogenated oil group (P < 0.001). In ghee oil group TG was significantly decreased, while Apo A had a significant increase (P < 0.01). Comparing with ghee group, liquid oil group had a significant reduction in HDL-C (P < 0.05).

## Discussion

This study indicated that liquid oil can generally reduce serum lipids levels when compared with hydrogenated oil. However, changes in serum lipids, except for TG reduction and Apo A enhancement, were not significant when ghee oil group was compared with hydrogenated oil group (serum HDL-C levels had an insignificant increase).

Epidemiological and intervention studies have strongly proposed that hydrogenated fat intake might enhance CVD risk, mainly by adversely affecting serum lipids profile.^28^, ^31^ TFAs are produced through the hydrogenation process of liquid vegetable oils. Considerable studies indicate that hydrogenated fat and/or TFAs could increase TC and LDL-C, and decrease HDL-C^32^–^36^ and some studies have shown serum TG elevation;^33^ however, the responsible mechanisms for these changes are complicated. It has been proposed that the serum lipid-raising effect of hydrogenated fat is due to either delayed LDL-C clearance or enhanced LDL-C production.^37^ Hayashi et al^38^ have reported that hydrogenated corn oil raised serum very low density lipoprotein cholesterol and LDL-C through the suppression of hepatic LDL receptor activity in hamsters. Another study also indicated^39^ that damaging the cholesterol catabolism is more responsible than decreasing its synthesis for higher serum TC seen by intake of high hydrogenated and saturated fat diets. But Kelley et al showed that diet containing cotton seed oil could not modify serum lipids including TC, TG, LDL-C, HDL-C, Apo A and Apo B in comparison with usual diet.^40^

As Asgari et al found,^41^ the average TFA contents in hydrogenated oils, liquid cooking and frying liquid oils produced in Iran were 35.2 ± 4.8%, 0.9 ± 0.3%, and 2.6 ± 0.8%, respectively. So, serum lipids modification by liquid oils seems reasonable in this study.

Ghee oil is an important dietary fat in India and other South Asian countries,^42^, ^43^ which contains high amounts of SFAs (about 59% of its whole fatty acids). SFAs, except for stearic acid, increase serum TC,^44^ and therefore, ghee oil, that are high in cholesterol and SFAs, are considered as harmful. On the other hand, ghee is a good source of oleic acid which is capable of protecting LDL-C particles from oxidation and prevents atherosclerosis.^45^, ^46^ Furthermore, according to Asgary et al, the average TFA content in ghee produced by Bakhtiari nomads (the kind of ghee that was used in this study) is 8.3 ± 0.7 which is 1.4 times less than the amount of existing TFA in hydrogenated oils.^41^

Kumar's study indicated that consumption of ghee in the diet even at high intakes does not increase serum lipids. A strong idea was also made to link the consumption of anhydrous milk fat such as ghee with increased risk of heart diseases.^47^ Kumar's study with experimental animals did not show any linking between ghee consumption to hypercholesterolemia and hyperlipidemia, which are considered to be risk factors for heart diseases. Interestingly, consuming increased levels of ghee reduced serum TC and TG levels.^47^ However, use of excess intake of ghee as a means for lowering serum TC is not recommended, but the study indicates that there is no reason for apprehension for consuming ghee in the diet, which is an age-old practice that is relished in Indian culinary.^47^ Mozaffarian et al obtained that substituting 8% of energy intake from TFA with SFA cause to decrease CVD by modifying TC/HDL-C ratio.^48^ So, it confirms the proper effect of ghee on serum lipid profile.

## Conclusions

Ghee was useful in modifying serum, including TG and HDL-C, and liquid oil consumption along with frying oil resulted in a general reduction in serum lipids. Therefore, it can be said that ghee might be effective on serum lipids modification in metabolic syndrome, but it should not be forgotten that ghee, which is traditionally made from milk fat, has high amounts of SFAs, and not forgetting that its production method should be carefully supervised.
